# The detection of cryptic *Plasmodium* infection among villagers in Attapeu province, Lao PDR

**DOI:** 10.1371/journal.pntd.0006148

**Published:** 2017-12-20

**Authors:** Moritoshi Iwagami, Sengdeuane Keomalaphet, Phonepadith Khattignavong, Pheovaly Soundala, Lavy Lorphachan, Emilie Matsumoto-Takahashi, Michel Strobel, Daniel Reinharz, Manisack Phommasansack, Bouasy Hongvanthong, Paul T. Brey, Shigeyuki Kano

**Affiliations:** 1 SATREPS project (JICA/AMED) for Parasitic Diseases, Vientiane, Lao PDR; 2 Department of Tropical Medicine and Malaria, Research Institute, National Center for Global Health and Medicine (NCGM), Shinjuku-ku, Tokyo, Japan; 3 Parasitology Laboratory, Institut Pasteur du Laos (IPL), Ministry of Health, Vientiane capital, Lao PDR; 4 Institut de la Francophone pour la Médecine Tropicale, Vientiane capital, Lao PDR; 5 Department of Social and Preventive Medicine, Université Laval, Quebec, Canada; 6 Laboratory Section, Center of Malariology, Parasitology and Entomology (CMPE), Ministry of Health, Vientiane capital, Lao PDR; 7 Center of Malariology, Parasitology and Entomology (CMPE), Ministry of Health, Vientiane capital, Lao PDR; 8 Institut Pasteur du Laos (IPL), Ministry of Health, Vientiane capital, Lao PDR; Universidad Peruana Cayetano Heredia, PERU

## Abstract

**Background:**

Although the malaria burden in the Lao PDR has gradually decreased, the elimination of malaria by 2030 presents many challenges. Microscopy and malaria rapid diagnostic tests (RDTs) are used to diagnose malaria in the Lao PDR; however, some studies have reported the prevalence of sub-microscopic *Plasmodium* infections or asymptomatic *Plasmodium* carriers in endemic areas. Thus, highly sensitive detection methods are needed to understand the precise malaria situation in these areas.

**Methodology/Principal findings:**

A cross-sectional malaria field survey was conducted in 3 highly endemic malaria districts (Xaysetha, Sanamxay, Phouvong) in Attapeu province, Lao PDR in 2015, to investigate the precise malaria endemicity in the area; 719 volunteers from these villages participated in the survey. Microscopy, RDTs and a real-time nested PCR were used to detect *Plasmodium* infections and their results were compared. A questionnaire survey of all participants was also conducted to estimate risk factors of *Plasmodium* infection. Numbers of infections detected by the three methods were microscopy: *P*. *falciparum* (n = 1), *P*. *vivax* (n = 2); RDTs: *P*. *falciparum* (n = 2), *P*. *vivax* (n = 3); PCR: *Plasmodium* (n = 47; *P*. *falciparum* [n = 4], *P*. *vivax* [n = 41], mixed infection [n = 2]; 6.5%, 47/719). Using PCR as a reference, the sensitivity and specificity of microscopy were 33.3% and 100.0%, respectively, for detecting *P*. *falciparum* infection, and 7.0% and 100.0%, for detecting *P*. *vivax* infection. Among the 47 participants with parasitemia, only one had a fever (≥37.5°C) and 31 (66.0%) were adult males. Risk factors of *Plasmodium* infection were males and soldiers, whereas a risk factor of asymptomatic *Plasmodium* infection was a history of ≥3 malaria episodes.

**Conclusions/Significance:**

There were many asymptomatic *Plasmodium* carriers in the study areas of Attapeu province in 2015. Adult males, probably soldiers, were at high risk for malaria infection. *P*. *vivax*, the dominant species, accounted for 87.2% of the *Plasmodium* infections among the participants. To achieve malaria elimination in the Lao PDR, highly sensitive diagnostic tests, including PCR-based diagnostic methods should be used, and plans targeting high-risk populations and elimination of *P*. *vivax* should be designed and implemented.

## Author summary

The Lao People’s Democratic Republic (Laos) is a country in the Greater Mekong Subregion. In Laos, the numbers of reported cases of malaria and deaths due to malaria have been gradually decreasing. Recently, the Lao government adopted a goal of eliminating malaria by 2030. To achieve this goal, we must understand the precise situation in each endemic area. With this background, we conducted a field survey in Attapeu, one highly endemic province, in 2015. We collected blood samples from 719 villagers, and most (98.1%, 705/719) had no fever (<37.5°C) at the time of survey. The samples were examined using three diagnostic methods: microscopy, rapid diagnostic test (RDT) and polymerase chain reaction (PCR). PCR revealed parasitemia in 6.5% of the villagers (47/719), and only one showed a fever (≥37.5°C) at the time of survey. The dominant species was *Plasmodium vivax* (87.2%, 41/47). Males and soldiers tended to be infected with malaria parasites whereas those with a history of multiple malaria episodes (≥3 times) tended to be asymptomatic malaria carriers. These findings suggest the need to implement targeted elimination plans for vivax malaria that include measures to protect males and soldiers, who are at high risk of malaria infection.

## Introduction

The malaria burden in the Lao People’s Democratic Republic (PDR) has gradually decreased thanks to the efforts of the Lao government and the support of partners such as the World Health Organization (WHO), and the Global Fund to Fight AIDS, Tuberculosis and Malaria [1]. In 2015, the malaria-associated mortality rate (number of deaths/100,000 population) was 0.03, which was lower than the target in Millennium Development Goal 6 (MDG 6) (<0.20) [2]. However, in the same year, the malaria-associated morbidity rate (annual parasite incidence [API]: number of cases/1,000 population) was 4.9, which was higher than the target in MDG 6 (<0.6). Now, the Ministry of Health (MOH) of the Lao PDR and the WHO have adopted the goal of eliminating malaria by the year 2030 [3, 4].

In central, provincial and district hospitals in the Lao PDR, malaria is typically diagnosed by microscopy, whereas rapid diagnostic tests (RDTs) are used as a sub-standard diagnostic method at locations in rural areas, such as health centers and selected villages with high malaria endemicity (API ≥10). Recently, highly sensitive methods, such as polymerase chain reaction (PCR) [5], ultra-sensitive PCR [6–8] and loop-mediated isothermal amplification (LAMP) [9–11] are becoming available and being used to detect low-level malaria infections in research bases in endemic areas. Such methods can detect sub-microscopic malaria infections and asymptomatic *Plasmodium* carriers in the endemic areas.

We conducted a malaria field survey of 10 villages in Xepon district, Savannakhet province in the Lao PDR, where malaria was highly endemic from August to September in 2013 (the rainy season) [5]. A nested PCR using dried blood samples from healthy villagers revealed that there were many asymptomatic *Plasmodium falciparum* carriers who could be considered to be parasite reservoirs or a cryptic malaria group. In most cases, the parasite densities among the villagers were below the microscopic threshold, i.e., sub-microscopic malaria cases. Interestingly, these parasite carriers were likely to be grouped within a family [5]. An Oxford research team also reported similar results but for many *P*. *vivax* carriers from the Thapangthong and Nong districts, which are also located in Savannakhet province, from March to July in 2015 (dry season to the beginning of the rainy season) [8].

**Fig 1:**
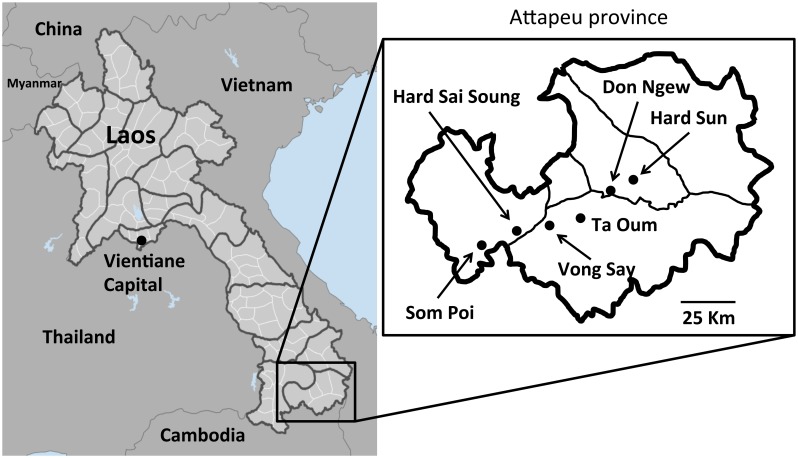
Study sites in Attapeu province, the Lao PDR. Don Ngew and Hard Sun villages are located in Xaysetha district (API = 31.2); Vong Say and Ta Oum villages are located in Phouvong district (API = 103.4); and Hard Sai Soung and Som Poi villages are located in Sanamxay district (API = 59.9). API, annual parasite incidence (according to the API data from 2013). Map of Laos (left), Data source: CIESIN, CIAT, GPW, Available from: http://sedac.ciesin.columbia.edu/gpw. Map of Attapeu province (right) was created by the authors.

Recently, there has been a gradual decrease in the number of reported cases of malaria in Savannakhet province thanks to the extensive efforts of the provincial health office and the support of several partners. In contrast, the number of reported cases in southern provinces, such as Salavan, Sekong, Attapeu and Champasak, remain high. According to the national data collected by the Center of Malariology, Parasitology and Entomology (CMPE) of the MOH in 2013, the API of Attapeu province was 37.8, which was the highest among all provinces in the Lao PDR. Therefore, in the present study, a cross-sectional field survey was conducted in Attapeu province, which borders Vietnam and Cambodia in the southern part of the Lao PDR (Fig 1), to investigate the prevalence of asymptomatic *Plasmodium* infections among the people in malaria-endemic villages, the distribution of the species of malaria parasites, and the risk factors of *Plasmodium* infections. We also evaluated the performance of our PCR technique in comparison to microscopy and RDTs.

## Methods

### Ethics statement

The research proposal was reviewed and approved by the National Ethics Committee for Health Research, Ministry of Health, Lao PDR (No. 049 NIOPH/NECHR) in 2014. Written informed consent was obtained from all of the participants prior to the interview and the collection of blood for the diagnosis of malaria. The guardians of child participants (<18 years old) consented to their participation.

### Sample collection

In May 2015, which was the beginning of rainy season, a cross-sectional field survey was conducted in the three districts that showed the greatest malaria endemicity in Attapeu province, Lao PDR: Xaysetha, Sanamxay and Phouvong (Fig 1). In 2013, the APIs of Xaysetha, Sanamxay and Phouvong were 31.2, 59.9 and 103.4, respectively. The populations of Xaysetha, Sanamxay and Phouvong were 32,888, 30,551 and 12,432, respectively, in 2014. The sample size of each district was calculated based on the malaria prevalences (API) in 2013 and the populations in 2014, with a confidence level of 95% and a confidence interval (CI) of 5%. The minimum sample sizes of Xaysetha, Sanamxay and Phouvong were 47, 87 and 143, respectively. Two malaria high endemic villages (strata 3; API ≥10) that were accessible by car were randomly selected in each district using village lists in district health offices.

Village leader or village health volunteer informed the villagers to join the malaria survey and asked not to go to their fields, forests or fishing on the day of the survey, if possible. The survey team was based at a temple, meeting place or health center in the villages and conducted an interview with and malaria blood test for the voluntary participants who came to us (convenience sampling). We accepted all voluntary participants who came to us regardless of their age, gender, occupation, ethnicity or health conditions. Information on demographics (age, gender, ethnicity, occupation, marital status, educational level), tympanic temperature, any current symptoms and signs (time first noticed), malaria treatment, number of previous malaria episodes (malaria history) and use of insecticide-treated bed nets was collected by using a questionnaire form.

An approximately 150–200 μL blood sample was collected from each participant by finger prick using a lancet. Three diagnostic methods were applied to detect *Plasmodium* infections in blood samples: microscopy (thin and thick blood smears), RDTs (SD Bioline Malaria Ag *Pf*/*Pv*, Standard Diagnostics, Inc., Gyeonggi-do, Republic of Korea) and PCR. The malaria RDTs were conducted on-site, whereas microscopy and the PCR were conducted at IPL in Vientiane after the survey. When *Plasmodium* infections were detected by the RDTs, an antimalarial medicine (Coartem; artemether + lumefantrine) was prescribed free of charge on-site by the staff members from the district hospital or health center that was conducting the survey in the village. For the PCR, blood samples were collected on filter papers (Whatman FTA Classic Cards, GE Healthcare Life Science, UK) in accordance with the manufacturer’s instructions.

### DNA extraction and real-time PCR

DNA was extracted from the dried blood spots on the filter papers with a QIAamp DNA Mini Kit (Qiagen, Hilden, Germany) in accordance with the manufacturer’s instructions. Six punched-out circles (3.175 mm [1/8 inch]) from the dried blood spot on the filter paper were used for DNA extraction, which was equivalent to 30–40 μL of whole blood. The extracted DNA was eluted with 50 μL of elution buffer in the kit and preserved until use at -30°C. To identify malaria parasite infection, a real-time nested PCR was conducted using a primer set that was reported in a previous study (S1 Table) [12, 13]. In the primary real-time PCR, a universal primer set for amplifying the partial *cytochrome b* gene on the mitochondrial genome of all human malaria parasites was used. In the secondary real-time PCR, primer sets that were specific for *P*. *falciparum* and *P*. *vivax* were used to detect the two species. The real-time PCR was performed using SsoAdvanced Universal SYBR Green Supermix (Bio-Rad Laboratory, Inc., USA) using 2 μL of the extracted DNA as a template, which was equivalent to 1.2–1.6 μL of whole blood. The primary PCR product was diluted 25 times with PCR-grade water, and 2 μL of the diluted primary PCR product was used as a template for the secondary real-time PCR. Serial diluted recombinant plasmid DNAs containing the *cyt b* region of *P*. *falciparum* or *P*. *vivax* were used as the positive control for each assay. PCR-grade water was used as the negative control for each assay. A sample was considered negative if there was no increase in the SYBR Green (fluorescent) signal after 35 cycles. The PCRs were performed independently, in triplicate. The sample was considered positive for *Plasmodium* DNA when positive results were obtained at least twice. As an external quality control for the PCR, some of the dried blood samples on the filter papers were sent to a laboratory in the National Center for Global Health and Medicine, Japan and examined by experienced researchers.

The sensitivity and specificity of microscopy and the malaria RDTs for the diagnosis of *P*. *falciparum* and *P*. *vivax* were calculated using the results of the real-time nested PCR as a reference.

### Statistical analysis

For bivariate analyses, the Chi-square test and Fisher’s exact test were used to evaluate an association between variables and *Plasmodium* infection. A *P* value less than 0.05 was considered statistically significant. Multivariate logistic regression analyses that adjusted for the effects of other variables were conducted to estimate the association between variables and *Plasmodium* infection and asymptomatic *Plasmodium* infection using SPSS version 18.0 (SPSS INC., Chicago, IL, USA). All variables with a P-value of 0.20 from univariate analysis were entered into a multivariate logistic regression analysis. Multicollinearity among all independent variables was tested before logistic regression.

Asymptomatic *Plasmodium* infection was defined by the following criteria: *Plasmodium* DNA was positive by PCR, tympanic temperature was less than 37.5°C (no fever) at the time of the survey and no history of any subjective symptoms and signs at the time of the survey and in the preceding 14 days.

## Results

### Socio-demographic data of the participants

A total of 719 villagers (male, n = 336; female, n = 383) participated in this survey (Table 1). The socio-demographic data of the participants are summarized in Table 2 and S2 Table. Most of the adult participants (n = 472; 65.6%) were farmers, and 160 of the participants (22.3%) were students. The study population included two major ethnic groups: Lao Loum (n = 189; 26.3%) and Lao Therng (n = 529; 73.6%). More than half of the participants (n = 410; 57.0%) had never been to primary school or had dropped out of primary school before graduation.

**Table 1:** The numbers of participants in the three districts of Attapeu province.

District	Village Name (latitude and longitude)	Date of Survey (Year, Month, Day)	Population of villages (2014)	No. of Participants	
				Male	Female
Xaysetha	Don Ngew	2015 May 11	903	63	69
	(N 14° 44’ 46.7”, E 106° 57’ 15.0”)				
	Hard Sun	2015 May 12	1,053	50	70
	(N 14° 47’ 45.0”, E 107° 01’ 06.7”)				
Phouvong	Vong Say	2015 May 13	431	44	63
	(N 14° 39’ 45.0”, E 106° 41’ 57.7”)				
	Ta Oum	2015 May 14	330	47	57
	(N 14° 40’ 53.2”, E 106° 49’ 36.0”)				
Sanamxay	Hard Sai Soung	2015 May 15	280	39	63
	(N 14° 38’ 36.4”, E 106° 34’ 04.7”)				
	Som Poi	2015 May 16	1,020	94	60
	(N 14° 33’ 29.3”, E 106° 27’ 59.3”)				
Total			4,017	337	382

**Table 2:** The socio-demographic data of the participants (N = 719).

Variable		Number	%
Gender	Female	383	53.3
	Male	336	46.7
Age (years)	Median ± SD (Range)	24 ± 16.6 (0–90)	
Occupation	Agriculture	472	65.6
	Student	160	22.3
	Child	54	7.5
	Trader	2	0.3
	Soldier	10	1.4
	Housewife	13	1.8
	Teacher	8	1.1
Education	No education or not completed*	410	57.0
	Primary school	240	33.4
	Secondary school	47	6.5
	High school	22	3.1
Marital status	Never married	288	40.1
	Married	425	59.1
	Divorced	4	0.6
	Widowed	1	0.1
	No data	1	0.1
Religion	Buddhism	234	32.5
	Christianity	3	0.4
	Traditional Animism	481	66.9
	Other	1	0.1
Ethnicity	Lao Loum	189	26.3
	Lao Therng	529	73.6
	Lao Soung	0	0.0
	No data	1	0.1
District	Xaysettha	252	35.0
	Phouvong	211	29.3
	Sanamxay	256	35.6

*Not completed, Dropped out of primary school before graduation.

### *Plasmodium* prevalence by the three diagnostic methods

Overall, *Plasmodium* DNA was detected in 47 (6.5%) participants by the PCR (S3 Table). Three of them were also detected by microscopy: *P*. *falciparum* (n = 1; 0.1%) and *P*. *vivax* (n = 2; 0.3%), and 5 of them were also detected by the RDTs: *P*. *falciparum* (n = 2; 0.3%) and *P*. *vivax* (n = 3; 0.4%) including the individuals who were microscopy-positive. Including all diagnostic tests, 5 of the 47 participants were *P*. *falciparum*, 41 of the 47 participants were *P*. *vivax* and 1 of the 47 participants was mixed infection with *P*. *falciparum* and *P*. *vivax*. Using the results of the real-time PCR as a reference, the sensitivity of microscopy for detecting *P*. *falciparum* and *P*. *vivax* was calculated as 16.7% (95% CI -13.2–46.5) and 4.7% (95% CI -1.6–10.9), respectively (Table 3). The sensitivity of the RDTs for *P*. *falciparum* and *P*. *vivax* was 33.3% (95% CI -4.4–71.1) and 7.0% (95% CI -0.6–14.6), respectively. The specificity of microscopy and the RDTs was 100.0% for both *P*. *falciparum* and *P*. *vivax*. With regard to gender, 41 of the 336 male participants were *Plasmodium*-positive, whereas only 6 of the 383 female participants were *Plasmodium*-positive (S4 Table).

**Table 3:** The sensitivity and specificity of microscopy and the RDT in comparison to real-time PCR.

	Real-Time PCR		
		Sensitivity (95% CI)	Specificity (95% CI)
Microscopy	*P*. *falciparum*	16.7% (-13.2–46.5)	100.0% (100.0–100.0)
	*P*. *vivax*	4.7% (-1.6–10.9)	100.0% (100.0–100.0)
RDT	*P*. *falciparum*	33.3% (-4.4–71.1)	100.0% (100.0–100.0)
	*P*. *vivax*	7.0% (-0.6–14.6)	100.0% (100.0–100.0)

RDT, rapid diagnostic test; CI, confidence interval.

The positive rate of the male participants (12.2%) was significantly higher than that of the female participants (1.6%) (*P* < 0.001). The positive rate of soldiers (50%) was the highest among the occupations (S4 Table). The prevalence of *Plasmodium* infections was heterogeneous among the districts: Xaysettha 0.40 (95% CI -0.38–1.18), Phouvong 7.11 (95% CI 3.61–10.60) and Sanamxay 12.11 (95% CI 8.09–16.13) (Table 4).

**Table 4:** Prevalence of *Plasmodium* infections.

Place	No. of Participants	No. Positive	Prevalence (95% CI)
Xaysettha District	252	1	0.40 (-0.38–1.18)
Don Ngew Village	132	0	0.00
Hard Sun Village	120	1	0.83 (-0.82–2.48)
Phouvong District	211	15	7.11 (3.61–10.60)
Vong Say Village	107	8	7.48 (2.41–12.54)
Ta Oum Village	104	7	6.73 (1.83–11.63)
Sanamxay District	256	31	12.11 (8.09–16.13)
Hard Sai Soung Village	102	5	4.90 (0.64–9.16)
Som Poi Village	154	26	16.88 (10.90–22.87)

CI, confidence interval.

### Clinical data of the participants

**Fig 2:**
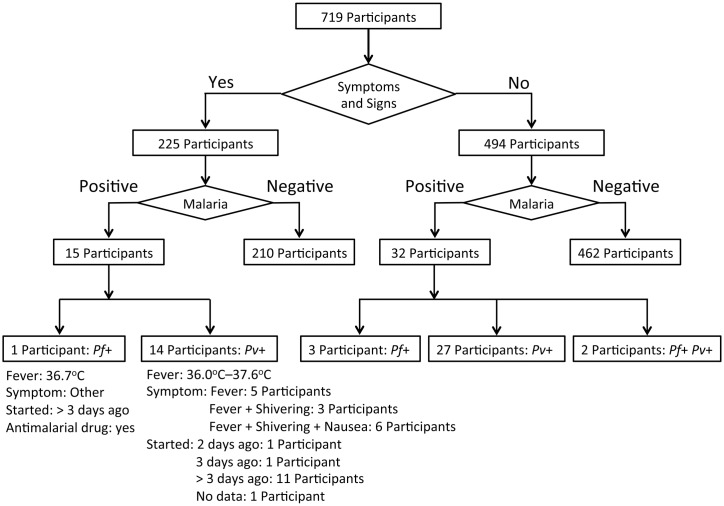
Flowchart summary of malaria screening based on the clinical and PCR data.

A flowchart summary of malaria screening based on the clinical and PCR data is shown in Fig 2. The average body temperature of the participants was 36.8°C (34.8°C–39.6°C), and only 14 participants (14/719: 1.9%) had a fever (>37.5°C) at the time of the survey. The average body temperature of the 47 PCR-positive participants was 36.7°C (36.0°C–37.6°C). A summary of the socio-demographic and clinical data of the 47 PCR-positive participants is shown in S3 Table. Only one of the 47 PCR-positive participants had a fever (>37.5°C) at the time of the survey. This febrile participant (ID: PT-015, 8 years of age, female) showed negative results for the blood smear and RDTs but was found to be infected with *P*. *vivax* using the real-time nested PCR. The fever started 2 days before the survey. This girl had used an insecticide-treated bet net while sleeping. The average age of all participants was 26.4 years (0–90 years, median age: 24 years), whereas that of the 47 PCR-positive participants was 29.0 years (6–75 years, median age: 25.0 years). Fifteen of the 47 PCR-positive participants answered that they had some symptoms or signs of health problems, such as fever, shivering and nausea, but only one participant had a fever (>37.5°C) at the time of the survey (S3 Table). Eleven of these 15 participants answered that the symptoms or signs started more than 3 days before the survey. In contrast, 32 of the 47 PCR-positive participants had no symptoms or signs of health problems at the time of and the preceding 14 days before the survey (S3 Table).

### Risk factors associated with *Plasmodium* infections

Results of bivariate analyses to estimate an association between variables and *Plasmodium* infections are shown in S4 Table. Risk factors associated with *Plasmodium* infections were estimated by multiple logistic regression analyses. Three variables were independently associated with *Plasmodium* infections: being male (AOR: 6.04, 95% CI: 1.35–27.10), Soldier (AOR: 28.58, 95% CI: 2.89–282.60), and being Lao Loum (AOR: 2.71 95% CI: 1.12–6.59) (Table 5). The risk factor associated with asymptomatic (cryptic) *Plasmodium* infections was a history of 3 or more malaria episodes (multiple malaria infections) (Model 1, AOR: 12.66, 95% CI: 1.21–132.00) (S5 Table).

**Table 5:** Multivariate logistic regression analyses to identify risk factors for *Plasmodium* infections.

Status		Univariate analysis			Multivariate analysis		
		OR	95% CI	*p*	AOR	95% CI	*p*
Village	Others	1.00			-		
	Som Poi	5.26	2.87–9.65	<0.001	-	-	-
Gender	Female	1.00			1.00		
	Male	8.71	3.65–20.80	<0.001	6.04	1.35–27.10	0.019
Occupation	Others	1.00			1.00		
	Soldier	15.88	4.42–57.00	<0.001	28.58	2.89–282.60	0.004
Education	Lower than High School	1.00			-		
	Equal to or higher than High School	4.59	1.61–13.0	0.004	-	-	-
Religion	Not Buddhism	1.00			-		
	Buddhism	2.76	1.52–5.02	0.001	-	-	-
Ethnicity	Lao Theung	1.00			1.00		
	Lao Loum	3.85	2.11–7.03	<0.001	2.71	1.12–6.59	0.027
ITBN use	Yes	1.00			-		
	No	0.49	0.18–1.31	0.156	-	-	-
Malaria past episode	≤ 2 times	1.00			1.00		
	≥ 3 times	3.35	1.48–7.61	0.004	2.35	0.93–5.96	0.071

OR, odds ratio; CI, confidence interval; AOR, adjusted odds ratio; ITBN, insecticide-treated bed net.

Multivariate analysis was adjusted for gender, occupation, ethnicity and past malaria episodes.

## Discussion

In the present study, all 719 participants resided in villages in the districts of Attapeu province that showed high malaria endemicity in the Lao PDR. We found 47 PCR-positive *Plasmodium* infections among the participants (6.5%), most (31/47, 66.0%) of whom were adult males (≥18 years). The national malaria data, which were summarized by the CMPE in 2015, revealed that *P*. *falciparum*, *P*. *vivax* and mixed infection accounted for 40%, 58% and 2% of malaria infections, respectively. The national data were based on the results obtained by microscopy (in hospitals) and RDTs (in health centers or villages) through the passive detection of symptomatic cases. In the present study, the proportions of *P*. *falciparum* (n = 2; 40%) and *P*. *vivax* (n = 3; 60%) infections detected by the RDTs were consistent with the national data from 2015.

However, the PCR analysis revealed that there were many *P*. *vivax* infections (41 *P*. *vivax* mono-infections and 2 *P*. *falciparum* and *P*. *vivax* mixed infections) among the participants. This finding suggests that malaria infections, particularly *P*. *vivax* infections, which can only be detected by microscopy or RDTs, represent the “tip of the iceberg”. Thus, a large portion of the malaria parasite population cannot be detected by the standard (microscopy) or sub-standard (RDTs) methods. To achieve the goal of eliminating malaria in the Lao PDR by 2030, a highly sensitive malaria detection method, such as PCR, should be implemented to detect the large portion of hidden malaria infections to prevent them from acting as reservoirs for the next outbreak of malaria.

In the present study, the logistic regression analyses revealed that the risk factors of *Plasmodium* infections were, being male, Soldier, and Lao Loum (Table 5). Moreover, the PCR revealed that 68.1% (32/47) of the participants with *Plasmodium* infections were asymptomatic or cryptic *Plasmodium* carriers (75% (3/4) of the *P*. *falciparum* infections, 65.9% (27/41) of the *P*. *vivax* infections, 100.0% (2/2) of the mixed infections with *P*. *falciparum* and *P*. *vivax*) in Attapeu province. The risk factor of cryptic *Plasmodium* infections was a history of 3 or more malaria episodes (S5 Table). Although male and soldier were the risk factors of *Plasmodium* infections, they were not the risk factors of cryptic *Plasmodium* infection. This might be associated with small sample size of the soldiers (only 10) or bias of the sample collection in this study (not a random sampling but a convenience sampling). Therefore, further investigations are requisite to understand the risk factor of soldier and male in Lao PDR. The prevalence of *Plasmodium* infection in Som Poi village was the highest among the 6 villages. Som Poi village is located near the border between the Lao PDR and Cambodia (Fig 1), and most residents were Lao Loum (98.7% 152/154), whose religion was Buddhism. The high prevalence of malaria in Som Poi village might be associated with their lifestyle in the village, environment including *Anopheles* mosquitoes or human migration between the Lao PDR and Cambodia.

There would be several reasons for this situation. In general, the adult populations in areas of high malaria endemicity might have acquired certain levels of immunity against malaria due to the frequent contraction of malaria parasites during their lives. Thus, the asexual growth of *Plasmodium* parasites in their blood would be suppressed, and the parasite might be maintained at a lower density, which could be lower than the threshold of detection by microscopy or rapid diagnostic testing. Indeed, the logistic regression analyses revealed that a history of multiple *Plasmodium* infections (>3 times) was significantly associated with the cryptic *Plasmodium* infections (S5 Table). Similar data was also reported from Cambodia [14]. Therefore, for effective control and elimination of malaria in the study area, special attention to and interventions for these high-risk populations are necessary. For example, active case detection for *Plasmodium* infection by highly sensitive methods such as PCR, LAMP or ultra-sensitive RDTs should be performed in high endemic villages or high-risk populations, such as males, soldiers or male farmers who stay overnight in the forest.

In the present study, 12.2% (41/336) of the males and 1.6% (6/383) of the females were infected with *Plasmodium* parasites. This discrepancy is probably associated with the occupational activities of the males. Most of the adult participants (65.6%, 472/719) in the present study were farmers. Both male farmers (205) and female farmers (267) worked in the fields, but only the males worked and sometimes stayed overnight in the forests with/without bed nets for the purpose of collecting food and logging. Another high-risk population was soldiers, who also work and stay overnight in the forest while training or on patrols, especially along the border of the country. In this sense, soldiers can be categorized as mobile and migrant populations that are considered to be high-risk populations for malaria [15]. In fact, 50% (5/10) of soldiers were infected with *P*. *vivax*, and 4 of the 5 soldiers had no symptoms at the time of the survey or in the preceding few days before the survey. Staying in the forest in the Greater Mekong Subregion is also considered to be associated with a high risk of malaria infection [16–21]. The difference between the prevalence of malaria among males and females has also been reported from the Thailand-Myanmar border, Cambodia and Ethiopia [22–24].

According to previous studies in low-transmission settings, the protective natural immunity to infection by *P*. *vivax* and *P*. *falciparum* is not understood [25]. However, there was a higher proportion of sub-microscopic and asymptomatic *P*. *vivax* infections in low-transmission settings, such as Cambodia, the Thailand-Myanmar border, Vietnam [26], the China-Myanmar border [27], Indonesia, Brazil [28], Colombia [29], Solomon Islands [30] and Ethiopia [31]. Our finding confirmed the situation in other low-transmission settings or pre-elimination settings in Southeast Asia, the Western Pacific, South America and Africa.

Another possible reason for the many *P*. *vivax* infections involves the treatment regimen that is administered for vivax malaria in the Lao PDR. The current guidelines recommend Coartem (artemether + lumefantrine) as the first-line drug for the treatment of vivax malaria, whereas previously, chloroquine had been recommended [1]. These antimalarial drugs can clear the asexual stage of *P*. *vivax* from the bloodstream of a patient. However, neither Coartem nor chloroquine can kill hypnozoites (a dormant stage of *P*. *vivax*) in human liver cells, which cause relapse. Presently, 8-aminoquinoline (or primaquine) is the only drug that shows efficacy against hypnozoites in the liver cells. A complete radical cure of vivax infection requires a 14-day course of primaquine [32]. However, although primaquine is included in the National Strategic Plan for Malaria Control and Elimination in the Lao PDR 2016–2020, availability of the drug is currently limited in hospitals in the 5 southern provinces because it causes acute hemolytic anemia among people with glucose 6-phosphate dehydrogenase (G6PD) deficiency. G6PD, which is one of the glycolysis enzymes in erythrocytes, protects erythrocytes from oxidative stress [33–35]. If the G6PD activity is lower than normal due to oxidative stress—such as the oxidative stress caused by taking primaquine—the erythrocytes will suddenly rupture and the patient will develop acute hemolytic anemia.

Although the prevalence of G6PD deficiency among populations in several countries—including the Greater Mekong Subregion—has been reported, the prevalence of G6PD deficiency among the Lao population is unknown [36]. Recently, the G6PD enzyme activity test (CareStart G6PD, Access Bio, Inc., Somerset, NJ, USA) and primaquine have become available at provincial and district hospitals in the 5 southern provinces in the Lao PDR. However, most of the vivax malaria patients in the Lao PDR reside in rural areas that are far from these hospitals. In such areas, malaria patients are likely to go to the nearest health center at which patients suspected of having malaria are diagnosed by malaria RDTs and treated with Coartem for both falciparum and vivax malaria. However, primaquine cannot be prescribed for vivax malaria patients at the health centers because in the treatment guideline of malaria in the Lao PDR, not only the G6PD test but also hematocrit and hemoglobin tests are required before prescribing primaquine for vivax malaria patients. Hematocrit and hemoglobin tests are not currently available at the health center level in the Lao PDR. Therefore, staff members at the health centers in the 5 southern provinces ask vivax malaria patients to go to district or provincial hospitals for radical treatment of vivax malaria, but many of them hesitate to go to these hospitals because they are far from their residence, i.e. they have to spend a lot of time and money on transportation. Thus, coverage with primaquine treatment for vivax malaria patients is still limited in the Lao PDR, and some vivax malaria patients may experience relapse several times (even within the same year). If the Lao MOH invests in facilities to provide blood tests such as hematocrit and hemoglobin at the health center level or simplify the treatment guideline for vivax malaria, elimination of vivax malaria would be accelerated.

The present study was conducted in May, which is the beginning of the rainy season in the Lao PDR. The predominance of *P*. *vivax* infection among the participants might have been influenced by this study period. According to a report from the CMPE in 2009–2015, the peak malaria incidence occurred between July to October in the Lao PDR. Generally, the prevalence of *P*. *falciparum* would be lower at the beginning of the rainy season because it is directly associated with the population density of *Anopheles* mosquitoes. If our field survey were conducted during July to October (the middle to the end of the rainy season), a greater number of *P*. *falciparum*-positive samples would have been detected in the same areas. In fact, in a previous study in Xepon district, Savannakhet province during August to September 2013, 78.8% (41/52) of *Plasmodium* infections comprised *P*. *falciparum* mono-infection [5], whereas in another study in Thapangthong and Nong districts, Savannakhet province during March to July 2015, 18.3% (32/175) of *Plasmodium* infections were *P*. *falciparum* mono-infection and 56.6% (99/175) were *P*. *vivax* mono-infection [8].

The current malaria elimination program in the Lao PDR only targets symptomatic cases. However, it has been shown that mosquitoes that feed on blood from people with sub-microscopic *Plasmodium* infections can transmit malaria [37, 38]. Thus, sub-microscopic *Plasmodium* carriers can contribute to malaria transmission [39, 40]. This fact must be kept in mind if the Lao PDR is to reach its ambitious goal of eliminating malaria by the year 2030.

Fever is one of the major symptoms of malaria. However, in the present study, only one of the 47 PCR-positive participants had a fever (>37.5°C) at the time of the survey. A similar result was reported from Africa. In low and unstable malaria transmission areas in Kenya, fever was a sensitive indicator of malaria only in children (<5 years) but not in older children and adults [41]. Thus, for screening malaria patients in low-transmission areas, fever may not be a useful indicator with which to detect malaria patients.

In the present study, there was no association between the use of insecticide-treated bed nets and *Plasmodium* infections. However, the villagers’ answers regarding the question about bed net use might be biased because some of our interviewers were the ones who distributed the bed nets to the villagers and asked them to use the nets when they sleep. Thus, some (or many) villagers were likely to answer “yes” to the interviewers even though they did not use the bed net at all or did not always use it.

One limitation of the present study was that we did not collect a history of sleeping away from home (e.g., sleeping in a farming hut) or in the forest or of working in the forest. Previous studies in the Greater Mekong Subregion including the Lao PDR showed that a history of sleeping in a farming hut or in the forest was associated with *Plasmodium* infections [8, 42, 43]. Forest workers in other Greater Mekong Subregion countries were also reported to be a high-risk group for malaria infections [16–21]. Nevertheless, if farmers use insecticide-treated bed nets properly while they sleep in the farming huts, *Plasmodium* infection might be preventable in the Lao PDR [44].

Another limitation of the present study was the exclusion of hard-to-access villages from the study area. Therefore, the villages selected in this study might not represent a complete picture of the districts. If a malaria survey is conducted in such hard-to-access villages in Attapeu province, there might be many malaria patients and asymptomatic *Plasmodium* carriers because in such villages, it is difficult for the villagers to access health care services even they have health problems. Thus, if there is a malaria patient or asymptomatic *Plasmodium* carrier and *Anopheles* mosquitos in one of these villages, the transmission would continue within the village, especially in the patient’s household and among the neighbors.

### Conclusion

The present PCR examination revealed 47 asymptomatic *Plasmodium* infections (6.5%) among the 719 voluntary participants from malaria-endemic villages in Attapeu province in May 2015. Most of them (66.0%; 31/47) were adult males (≥18 years old), and the dominant parasite species was *P*. *vivax*. Males and soldiers were associated with *Plasmodium* infections whereas a history of 3 or more malaria episodes was associated with cryptic *Plasmodium* infections. Of the 32 asymptomatic *Plasmodium* carriers, 30 (93.8%) had infections that were below the detection threshold of microscopy, and 28 (87.5%) had infections that were below the detection threshold of the RDTs. To achieve the goal of malaria elimination in the Lao PDR by 2030, sensitive diagnostic methods—such as a PCR- or LAMP-based method—should be utilized, and plans targeting high-risk populations (males and soldiers) and the elimination especially of *P*. *vivax* should be designed and implemented.

## Supporting information

S1 Checklist STROBE checklist. (DOC)

S1 Table List of PCR primers. (XLSX)

S2 Table Socio-demographic data of the participants in each village. (XLSX)

S3 Table Summary of socio-demographic and clinical data of the 47 participants PCR-positive for malaria. (XLSX)

S4 Table Bivariate analyses to identify an association between variables and *Plasmodium* infections. (XLSX)

S5 Table Multivariate logistic regression analyses to identify risk factors for cryptic *Plasmodium* infections. (XLSX)
